# Effect of Drying Methods on the Steroidal Alkaloid Content of Potato Peels, Shoots and Berries

**DOI:** 10.3390/molecules21040403

**Published:** 2016-03-25

**Authors:** Mohammad B. Hossain, Nigel P. Brunton, Dilip K. Rai

**Affiliations:** 1 Department of Food Biosciences, Teagasc Food Research Centre, Ashtown, D15 DY05 Dublin, Ireland; dilip.rai@teagasc.ie; 2 School of Agriculture and Food Science, University College Dublin, D04 V1W8 Dublin, Ireland; nigel.brunton@ucd.ie

**Keywords:** potato, α-solanine, α-chaconine, drying, abiotic stress, UPLC-MS/MS

## Abstract

The present study has found that dried potato samples yielded significantly higher levels of steroidal alkaloids such as α-solanine and α-chaconine than the corresponding fresh samples, as determined by the UPLC-MS/MS technique. Among the drying techniques used, air drying had the highest effect on steroidal alkaloid contents, followed by freeze drying and vacuum oven drying. There was no significant difference between the freeze dried and vacuum oven dried samples in their α-chaconine contents. However, freeze dried potato shoots and berries had significantly higher α-solanine contents (825 µg/g dry weight (DW) in shoots and 2453 µg/g DW in berries) than the vacuum oven dried ones (325 µg/g dry weight (DW) in shoots and 2080 µg/g DW in berries). The kinetics of steroidal alkaloid contents of potato shoots during air drying were monitored over a period of 21 days. Both α-solanine and α-chaconine content increased to their maximum values, 875 µg/g DW and 3385 µg/g DW, respectively, after 7 days of drying. The steroidal alkaloid contents of the shoots decreased significantly at day 9, and then remained unchanged until day 21. In line with the potato shoots, air dried potato tuber peels also had higher steroidal alkaloid content than the freeze dried and vacuum oven dried samples. However, a significant decrease of steroidal alkaloid content was observed in air dried potato berries, possibly due to degradation during slicing of the whole berries prior to air drying. Remarkable variation in steroidal alkaloid contents among different tissue types of potato plants was observed with the potato flowers having the highest content.

## 1. Introduction

**Figure 1:**
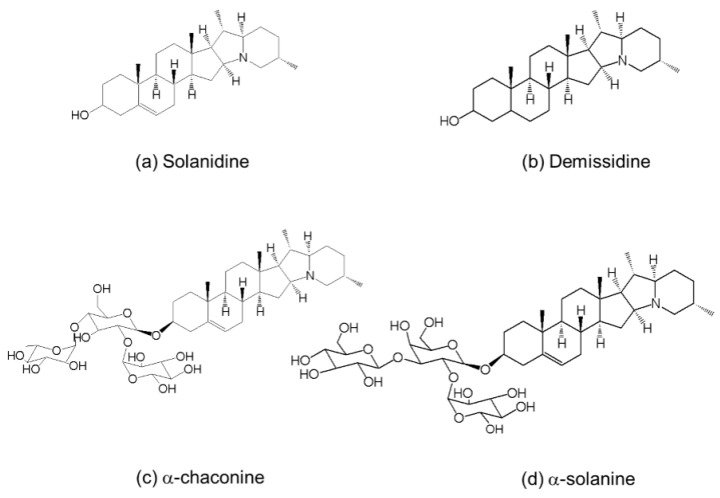
Chemical structures of glycolkaloids in potato peels.

A high volume of potato peels are generated in the potato processing industries as a byproduct, which has low or no value. These by-products are generally discarded in landfills with accompanying negative effect on environment or used as low value animal feed [1]. Currently food processors, government bodies and downstream processors are aware of the commercial potentials of the high volume by-products such as potato peels. However, challenges remains for finding the best possible way to maximise benefit while minimising cost and time of the utilization process. Potato peels are rich source of steroidal alkaloids (Figure 1) which are well known for their toxicity for human consumption in considerably high concentration (>1 mg/g dry weight sample) [2,3]. However, recent studies have demonstrated that these compounds also possess beneficial properties such as anticancer [4] and anti-inflammatory effects [5] depending on dose and conditions of use [6]. Particularly, α-chaconine has demonstrated similar anticancer activity to that of the commercial anticancer drugs such as tamoxifen [5]. Although the anticancer properties of solanum steroidal alkaloids have been well studied, the necrotic mode of action of these compounds against cancerous cells makes them unsuitable for use as an anticancer drug [5]. In a recent development in this area, apoptotic compounds have been synthesized from a steroidal compound, lanosterol [7]. Therefore, it has been hypothesized that chemical modification of potato steroidal alkaloids would generate novel apoptotic anticancer compounds which might show enhanced anticancer property possibly equivalent or higher to tamoxifen. In order to carry out such chemical modifications, which include a considerable number of sequential reactions, a large quantity of solanum steroidal alkaloid is required as a substrate. This has led to investigations of the different potato plant parts along with highly abundant potato peels for their steroidal alkaloid content in order to obtain high quantity of steroidal alkaloid using lowest possible amount of solvent, time and energy.

Potato plant parts (peels, shoots and fruits) are highly perishable items with high content of moisture and carbohydrate that facilitates microbial growth. The biologically active compounds in potato peels are also prone to enzymatic, environmental and microbial degradation during storage. In order to avoid these problems, drying of these plant parts could be an easy and inexpensive alternative to freezing. In addition, the dried samples are convenient to reduce particle size which in general enhances extraction of the target compounds. To the best of our knowledge, there has been no study on the effect of drying on the steroidal alkaloid content of potato plant parts. Therefore, the present study has investigated the effect of drying on the steroidal alkaloid content of the three most abundant parts of potato plant namely peels, shoots and fruits also known as berries. 

## 2. Results and Discussion

### 2.1. Distribution of Steroidal Alkaloids in Different Parts of Potato Plant

**Figure 2:**
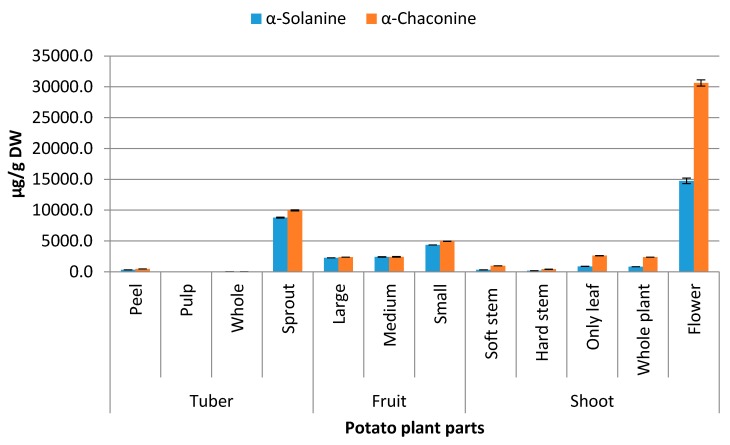
Distribution of steroidal alkaloids in different parts of potato plants (error bars represent standard deviation, *n =* 6).

Different potato plant parts showed a wide variation in their steroidal alkaloid content (Figure 2). The highest total steroidal alkaloid content was observed in potato flowers, followed by sprouts. These two plant parts being most vulnerable to biotic and abiotic stresses are very important for the plant for sexual and vegetative reproduction, respectively. This warrants a high level of protection in place for these two parts. Therefore, the contents of steroidal alkaloids, which are recognised as strong defence compounds [8] were expected to be higher in these tissues. In general, the intrinsic distribution of steroidal alkaloid content of potato plants was guided by this principle of vulnerability and importance even before the exposure to stresses although exposure of individual tissues to external stresses might also contribute to accumulation of the steroidal alkaloids irrespective of the tissue types. In line with this observation potato fruits, particularly the young ones, had the highest steroidal alkaloid content following the flowers and sprouts. The steroidal alkaloid contents of the fruits (also known as berries) were reduced significantly (*p <* 0.05) as the size of the berries increased (Figure 2). In fact, the small (diameter = 1 cm) berries had twice the steroidal alkaloid content than the large ones (diameter = 2 cm). The next level of steroidal alkaloids was observed in potato shoots, followed by potato tubers. The different parts of the shoot had steroidal alkaloid levels in the following decreasing order: leaves > soft stem > hard stem. Friedman and Dao [9] have also reported the same sequence while analysing the steroidal alkaloid content in different parts of the plant. However, contrary to Friedman and Dao’s work, the present study found potato berries, the small ones in particular, had higher steroidal alkaloid content than the stems and leaves. This difference might be attributed to cultivar and size of the berries. In tubers the peels had higher steroidal alkaloid content than the pulp. The ratio of total steroidal alkaloid content in potato flowers (45,391 µg/g DW) to potato tuber pulp (26 µg/g DW) was 1728, indicating the extent of variation among tissue types. Overall, the content of α-chaconine was higher than α-solanine in all tissue types. However, the ratio of α-chaconine to α-solanine had a wide variation among tissue types, ranging from 1.0 in medium sized potato berries to 3.1 in small stems.

The other tissue types with high α-chaconine ratio were leaves (2.98), flowers (2.08) and hard stem (2.45). Previous report on this ratio among different tissues of potato plant was 1.2 to 2.7 [9]. Since α-chaconine is more cytotoxic than α-solanine, the tissues with higher ratio of α-chaconine are more likely to induce toxic symptoms than their counterparts. These findings would have implications in the safety aspects of the tissues for consumption, as leaves and soft stems are consumed in some part of the world such as Bangladesh [10]. 

### 2.2. Changes in Steroidal Alkaloid Content during Air Drying of Potato Shoots

**Figure 3:**
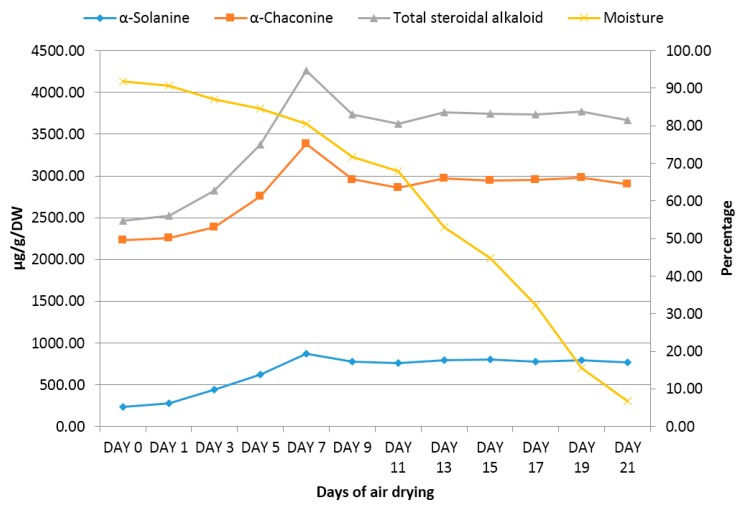
Kinetics of steroidal alkaloids during air drying of potato shoots (*n =* 6).

Potato shoots showed a remarkable increase of total steroidal alkaloid content during air drying reaching the maximum at day 7 (Figure 3). The total glycoalkaloid content in the samples collected at day 7 was 72.7% higher than the content in the samples at day 0. The rate of increase at early stage of drying was slow contributing only 14.3% increase at day 3. On the other hand, the samples showed 36.8% increase of total steroidal alkaloid content at day 5 while reaching the maximum (72.7%) at day 7. Gradual moisture loss during air drying might have triggered the stress response system possibly through ethylene signalling pathways of the plant which was responsible for the accumulation of the steroidal alkaloids [11]. The changes of steroidal alkaloid content during air drying was not due to the difference in extraction efficiency as the samples collected over the drying period were uniformly vacuum oven dried and milled (particle size approximately 0.2 mm) before extraction. There might be causes other than moisture stress response such as light and temperature for the changes in steroidal alkaloid contents during air drying. However, determination of the causes leading to the changes in steroidal alkaloid contents during air drying was not the objective of the paper. All steroidal alkaloid values reported in this manuscript were corrected for the moisture loss (%) of the respective samples during drying (Table 1).

Similar effect of air drying was observed in a separate study on a different class of stress response compounds such as polyphenols in Lamiaceae herbs [12]. To the best of our knowledge this paper is the first report on effect of drying on steroidal alkaloid contents in potato tissues. However, a number studies have documented the effect of light and temperature [13,14,15,16,17] which can also be categorised along with drying as abiotic stress on glycoalkaloid accumulation in potato tubers. After 5 days of exposure in the air drying environment the moisture level might be critically low (<85%) which might be responsible for rapid increase of the steroidal alkaloids. However, the samples of day 9 showed a marked decrease in the steroidal alkaloid content from 4260 µg/g DW (±SD = 120) at day 7 to 3733 µg/g DW (±SD = 60) at day 9. This was probably due to plant’s detoxification mechanism by enzymatic processes as steroidal alkaloids being stable compounds were unlikely to be degraded by ambient light and temperature. The very high levels of steroidal alkaloids at day 7 might have hampered plant’s normal cellular mechanisms leading to removal of excess alkaloids. The threshold of steroidal alkaloids seems dependant on tissue types as potato flowers and sprouts contain several times higher steroidal alkaloid content than potato shoots unaffected by detoxification mechanisms. When the moisture level was decreased below 70% at day 11 and onwards there was no further change in the steroidal alkaloid content. At this moisture level cellular processes might have slowed down or stopped to have further change in the steroidal alkaloid content. 

### 2.3. Effect of Different Drying Methods on Steroidal Alkaloid Content of Different Plant Parts

**Figure 4:**
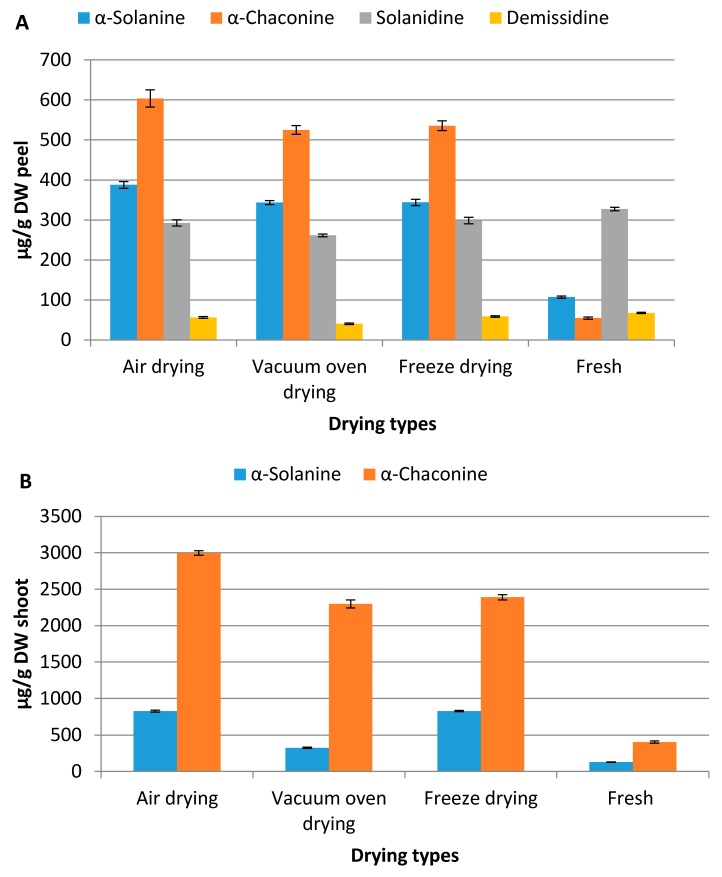
Levels of steroidal alkaloids in potato peel (**A**) and shoots (**B**) following different drying conditions (Error bars represent standard deviation, *n =* 6).

Significant increase of total steroidal alkaloid contents in industrial potato peels and shoots was observed due to drying. Among the drying methods applied, the highest levels of this group of biologically important compounds were measured in air dried samples followed by freeze drying and vacuum oven drying while the fresh samples had the lowest content (Figure 4).

The highest content in air dried samples was possibly due to stress response as mentioned earlier. Because air drying is a slow process, losing moisture while the tissues are still biologically active could be considered as stress for the tissues. The drying process during freeze drying is rapid and the drying conditions in this technique are not suitable for metabolic processes. Therefore, the increases of steroidal alkaloid contents in this drying method was possibly due to inactivation of degrading enzymes and higher extraction efficiencies in dried samples. However, significant degradation of α-solanine might have taken place during vacuum oven drying before the moisture level and temperature of the sample got unfavourable for metabolic processes. The other main glycosylated steroidal alkaloid α-chaconine seems more resilient to enzymatic degradations as this compound is the main line of defence for the plant [18,19].

Dried samples are usually brittle and get into smaller particle size than their fresh counterparts. This enhances the cell wall breakages, surface area of extraction and deeper penetration of the extracting solvent into the sample matrices. All these factors are known as the major contributing factors for extraction of target compounds from biological matrices.

**Figure 5:**
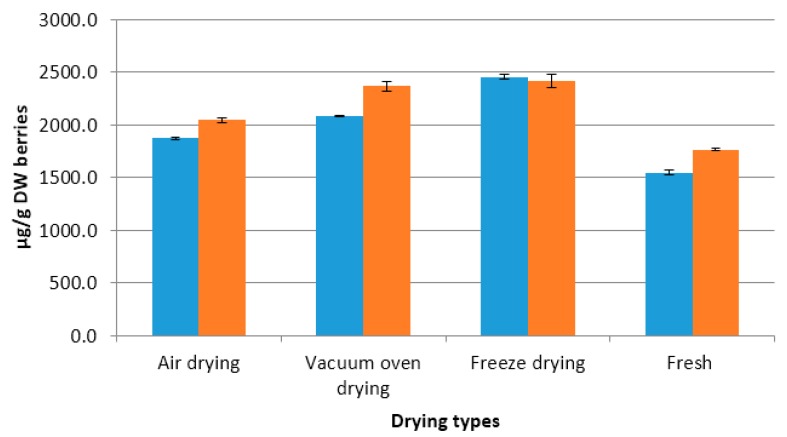
Levels of steroidal alkaloids in potato berries dried using different drying methods (Error bars represent standard deviation, *n =* 6).

Suhaj [20] suggested to use dried samples for extraction as some of the bioactive compounds might be unstable or degraded by enzymatic actions in fresh samples. In all drying processes, the intercellular spaces of tissues collapse, liberating more bioactive secondary metabolites such as steroidal alkaloids and polyphenols [21,22]. The fresh samples were ground into paste releasing active steroidal alkaloid degradation enzymes such as solaninase. Degradations of steroidal alkaloids by potato plant’s sap have been shown by Nikolic and Stankovic [23]. More intact cell wall structures and presence of degradation enzymes could be the reason for the lowest steroidal alkaloid content in the fresh samples. Meanwhile, in potato berries the air dried samples had lower contents than the other drying methods (Figure 5). This was because the berries were chopped into smaller pieces (2–3 mm in thickness) to facilitate air drying while in vacuum oven and freeze drying the berries were only halved and immediately put into the drying systems. Chopping might have helped in releasing steroidal alkaloid degrading enzymes lowering the content in air dried berries. 

In addition, the present study has observed that the peels from potato processing industries also had aglycone alkaloids (solanidine and demissidine) along with glycoalkaloids (α-solanine and α-chaconine) whereas all other samples had only the glycosylated steroidal alkaloids. The glycosylated alkaloids were possibly hydrolysed into aglycone alkaloids due to fermentation and microbial degradation during industrial processes and storage of the peels. However, the response of both aglycone and glycoalkaloids were similar to drying related stresses.

## 3. Experimental Section

### 3.1. Samples and Reagents

Potato plant parts (shoots, leaves, flowers and berries) of variety Cara have been collected from a field in Oak Park, Ireland. The age of the plants was 100 days. The plants were grown in a slightly acidic soil (pH 5.5) with average monthly air temperature of 13 °C, soil temperature (at 10 cm depth) of 15 °C and rainfall of 43 mm. Potato peel slurry was kindly provided by Largo Foods Limited (Meath, Ireland). The steroidal alkaloids such as α-solanine, α-chaconine, solanidine and demissidine were purchased from Extrasynthese (Genay Cedex, France). HPLC grade solvents methanol, water, formic acid and acetonitrile were purchased from Sigma-Aldrich (Wicklow, Ireland).

### 3.2. Drying of Fresh Potato Plant Parts

The potato plant parts were dried using one of the following drying methods: (a) drying at ambient temperature in a dark, well-ventilated room for three weeks (mean temperature 14 °C; mean relative humidity 10%); (b) drying in a vacuum oven (Gallenkamp, Leicestershire, UK) at 70 °C for 16 h in the vacuum of 600 mbar; (c) freeze-drying in a Cuddon freeze-drier, model no. FD80 (Cuddon Freeze Dry, Blenheim, New Zealand) at a temperature of −54 °C and a pressure of 0.064 mbar for 72 h. All the dried samples were immediately vacuum packed and kept in −20 °C prior to extraction and analyses. The film (75 micron thickness) of the vacuum pack pouches (Allfo vakuumverpackungen Hans Bresele KG, Waltenhofen, Germany) was composed of a mixture of polyamide (PA) and polyethylene (PE) with an oxygen and carbon dioxide permeability rate of 60 cm^3^/m^2^/24 h/atom and 180 cm^3^/m^2^/24 h/atom, respectively (23 °C, 75% RH). The water vapour permeability of the film was 2.7 g/m^2^/24 h at 23 °C and 85% RH. For the study of air drying kinetics, the samples were taken off from the drying conditions at intervals of 1, 3, 5, 7, 9, 11, 13, 15, 17, 19 and 21 days and freeze dried immediately as mentioned above. Fresh samples were considered as control. Moisture content the sample was measured by using the following equation:

(1) $$\mathrm{Moisture}\% =\frac{F-D}{F}\times 100$$

where F stands for fresh weight and D stands for dry weight when it reached a constant. Extraction and quantitation of the steroidal alkaloids were performed immediately after drying. 

### 3.3. Preparation of Extracts from Fresh Samples

Sample preparation was carried out following the procedure as described by Hossain *et al.* [12] with slight modifications. The potato plant parts were chopped into small pieces of approximately 0.5 cm^2^ and milled into semi-paste by a kitchen blender (Kenwood Limited, London, UK). An amount fresh weight of the semi-paste plant parts which was equivalent to 1.0 g dry weight of the respective sample calculated from their moisture content was immediately mixed with 25 mL of methanol (100%) and homogenised with Omni-prep multisample homogeniser (Omni International, Kennesaw, GA, USA) at 24,000 rpm. The homogenised sample suspension was shaken overnight with a V400 Multitude Vortexer (Alpha Laboratories, North York, ON, Canada) at 1500 rpm at room temperature. The sample suspension was then centrifuged at 2000 *g* for 15 min and immediately filtered through 0.22 µm polytetrafluoroethylene (PTFE) filters. The extracts were kept in −20 °C for subsequent analysis.

### 3.4. Preparation of Extracts from Dried Plant Parts

The dried potato plant parts were milled directly without chopping by a kitchen blender. The powdered plant parts (1.0 g) were mixed with 25 mL of 100% methanol and homogenised with Omni-prep multisample homogeniser at 24,000 rpm. The homogenised sample suspension was shaken overnight with a V400 Multitude Vortexer (Alpha Laboratories) at 1500 rpm at room temperature. The sample suspension was then centrifuged for 15 min at 2000 *g* and filtered through 0.22 µm PTFE filters. The extract was kept in −20 °C for subsequent analysis of steroidal alkaloids. 

### 3.5. Identification and Quantification of Steroidal Alkaloids in Potato Peel by Ultra-Performance Liquid Chromatography Coupled with Tandem Mass Spectrometry

Steroidal alkaloids were analysed using Waters Acquity (Waters Corporation, Milford, MA, USA) ultra-high performance liquid chromatography coupled with tandem mass spectrometry (UHPLC-MS/MS) using a validated method for potato steroidal alkaloids [24]. The recovery rate of the method ranged from 97% to 103% for four steroidal alkaloids analysed. The compounds were separated on a Waters Acquity BEH C18 column (50 × 2.1 mm, particle size 1.7 µm) using 0.5% formic acid in water (Solvent A) and a mixture of acetonitrile, 2-propanol, and formic acid in the ratio of 94.5:5:0.5 (solvent B) as described by Paul *et al.* [25]. The following stepwise gradient program was carried out: 0–1 min 10% B, 2–6 min 20.5% B, 7–9 min 30% B, 9.5 min 90% B and 10–11 min 10% B at a flow rate of 0.5 mL/min. The injection volume for all the samples was 5 μL. All the standards in the concentration ranging from 0.002 to 0.1 µg/mL for quantification purposes were dissolved in 80% methanol–20% water extract of sweet potato which does not contain steroidal alkaloids. Due to unavailability of negative potato matrices, sweet potato extract was considered as matrix for potato extract as they had similar protein, fat and carbohydrate contents. Samples for UHPLC-MS/MS analysis were prepared in (80% methanol in water) following the extraction described in the earlier Section 2.3. Multiple reaction monitoring (MRM) was used to quantify different steroidal alkaloids. The parameters for the MRM transitions shown in Table 2 were determined and optimised using the Waters integrated Intellistart™ software. The ionization source conditions were as follows: capillary voltage 3 kV, Cone voltage 30 V, extractor voltage 3 V, source temperature 120 °C, desolvation temperature 350 °C, desolvation gas flow 800 L/h, cone gas flow 50 L/h, and collision gas flow 0.10 mL/min. The MRM traces were acquired using the Waters MassLynx V4.1™ software (Waters Corporation) while the quantifications of the data were carried out using the Waters TargetLynx™ software (Waters Corporation).

Standard methanolic solutions of individual steroidal alkaloids were prepared in the concentration range of 0.1 µg/mL to 1.0 µg/mL. The data (area under the curve) acquired for respective masses were used to obtain the standard curve (R^2^ = 0.991). Matrix effect was minimized by dissolving the standards in a similar matrix to the sample matrix. Therefore, any ion suppression if it occurs would affect the standards and sample in a similar way. In addition, targeted analyses using multiple reactions monitoring (MRM) reduced the interferences from matrix and competing compounds.

### 3.6. Statistical Analysis

Analysis of variance (ANOVA) was carried out using the software STATGRAPHICS Centurion XVI (Statpoint Technologies, Inc., Warrenton, VA, USA). ANOVA test was carried out for all experimental runs to determine significant differences among treatments at α = 0.05 levels. 

## 4. Conclusions

Drying has significant effect on the steroidal alkaloid content of potato plant parts. The effect is more pronounced in air dried potato shoots and peels. However, berries had higher content of steroidal alkaloid when they were freeze dried. Minimal processing such as chopping, milling of fresh potato plant parts decreased the steroidal alkaloid levels in these tissues. A remarkable variation in the levels of steroidal alkaloids in different potato plant parts was observed with potato flower and tuber pulp having the highest and lowest content, respectively.

## Figures and Tables

**Table 1:** Moisture loss (%) of the samples during drying (Mean ± SD, *n =* 6).

Sample	Moisture Loss (%)		
	Freeze Drying	Air Drying	Vacuum Oven Drying
Potato peel	78 ± 0.6	75 ± 0.5	77 ± 1.1
Shoots	92 ± 1.0	90 ± 1.2	91 ± 1.0
Berries	85 ± 1.5	82 ± 0.5	83 ± 1.5

**Table 2:** MRM parameters for UHPLC-MS/MS data acquisition of steroidal alkaloids of potato peel

Compound	MRM Transitions	Dwell Time (ms)	Cone Voltage (V)	Collision Energy (eV)
α-Solanine	*m/z* 868.54➔*m/z* 722.30	0.042	98	79
α-Chaconine	*m/z* 852.53➔ *m/z* 706.75	0.042	94	76
Solanidine	*m/z* 398.24➔ *m/z* 147.42	0.042	62	42
Demissidine	*m/z* 400.37 ➔ *m/z* 118.68	0.042	74	72
